# Efficacy, safety, and cost-effectiveness of pegylated PEG-rhg-CSF in pediatric patients receiving high-intensity chemotherapy: results from a phase II study

**DOI:** 10.3389/fphar.2024.1419369

**Published:** 2024-07-17

**Authors:** Junting Huang, Jia Zhu, Lian Jiang, Jiaqian Xu, Xiheng Lin, Jian Chang, Xiaohong Zhang, Suying Lu, Feifei Sun, Juan Wang, Yi Que, Zhonglv Ye, Lihua Yang, Xiuli Yuan, Weisong Cai, Chuan Tian, Yanpeng Wu, Xiangling He, Yan-Lai Tang, Yizhuo Zhang

**Affiliations:** ^1^ Department of Pediatric Oncology, State Key Laboratory of Oncology in South China, Collaborative Innovation Center for Cancer Medicine, Sun Yat-Sen University Cancer Center, Guangzhou, China; ^2^ Department of Pediatrics, The Fourth Hospital of Hebei Medical University (Hebei Tumor Hospital), Shijiazhuang, China; ^3^ Department of Pediatric Oncology, The Fifth Affiliated Hospital of Guangzhou Medical University, Guangzhou, China; ^4^ Department of Pediatric Oncology, The First Bethune Hospital of Jilin University, Changchun, China; ^5^ Department of Hematology and Oncology, Guangzhou Women and Children’s Medical Center, Guangzhou, China; ^6^ Department of Children’s Medical Center, Affiliated Hospital of Guangdong Medical University, Zhanjiang, China; ^7^ Department of Pediatric Center, ZhuJiang Hospital of Southern Medical University, Guangzhou, China; ^8^ Department of Hematology and Oncology, Shenzhen Children’s Hospital, Shenzhen, China; ^9^ Department of Oncology, ShengJing Hospital of China Medical University, Shenyang, China; ^10^ Department of Pediatric Hematology and Oncology, Hunan Provincial People’s Hospital, Changsha, China; ^11^ Department of Pediatrics, The First Affiliated Hospital, Sun Yat-sen University, Guangzhou, China

**Keywords:** phase ii study, high-intensity chemotherapy, neutropenia, PEG rhG-CSF, pediatric cancer patients

## Abstract

**Background:**

High-intensity chemotherapy can cause life-threatening complications in pediatric patients. Therefore, this study investigated safety and efficacy of long-acting pegylated recombinant human granulocyte colony-stimulating factor (PEG-rhG-CSF; Jinyouli^®^) in children undergoing high-intensity chemotherapy.

**Methods:**

Treatment-naive patients received post-chemotherapy PEG-rhG-CSF as primary prophylaxis for two cycles. The primary endpoints were drug-related adverse events (AEs) and bone pain scores. Secondary endpoints included grade 3–4 neutropenia, duration of neutropenia recovery, absolute neutrophil count changes, febrile neutropenia (FN), reduced chemotherapy intensity, antibiotic usage, and AE severity. The cost-effectiveness of PEG-rhG-CSF was compared with that of rhG-CSF (Ruibai^®^).

**Results:**

Here, 307 and 288 patients underwent one and two PEG-rhG-CSF cycles, respectively. Ninety-one patients experienced drug-related AEs, primarily bone pain (12.7%). Moreover, Grade 3–4 neutropenia and FN were observed. Median FN durations were 3.0 days in both cycles. No drug-related delays were observed during chemotherapy. One patient experienced grade 4 neutropenia-induced reduction in chemotherapy intensity during cycle 2. In total, 138 patients received antibiotics. PEG-rhG-CSF exhibited superior cost-effectiveness compared to rhG-CSF.

**Conclusion:**

Our findings indicate that PEG-rhG-CSF is safe, efficient, and cost-effective in pediatric patients undergoing high-intensity chemotherapy, providing preliminary evidence warranting further randomized controlled trials.

## 1 Introduction

High-intensity chemotherapy has become the routine treatment for most pediatric patients with cancer (Wittman et al., 2006). However, neutropenia and febrile neutropenia (FN) are common and potentially life-threatening complications of this treatment in pediatric patients (Ammann et al., 2010; Lehrnbecher et al., 2023). Despite improved medical management, FN is associated with severe infection and may lead to reduced dose intensity, worsening clinical efficacy, economic burden, and even death (Crawford et al., 2004; Fortner et al., 2005; Pathak et al., 2015).

The International Pediatric Fever and Neutropenia Guideline Panel recommend the primary prophylactic use of recombinant human granulocyte colony-stimulating factor (rhG-CSF) or pegylated rhG-CSF (PEG-rhG-CSF) in pediatric patients receiving chemotherapy (Lehrnbecher et al., 2023). However, the short plasma half-life (approximately 3–4 h) of rhG-CSF necessitates daily subcutaneous injections, potentially affecting patient compliance (Liu et al., 2021). Pegfilgrastim (Neulasta^®^) is a once-per-cycle PEG-rhG-CSF with a long half-life (33.2–62.1 h) and high activity. This drug has been approved for pediatric use by the United States Food and Drug Administration (Zamboni, 2003; Hu et al., 2021). Although several pegfilgrastim biosimilars and the third-generation rhG-CSF efbemalenograstim alfa have been approved for clinical application, their approvals were primarily based on adult data (Harbeck et al., 2016; Glaspy et al., 2017; Waller et al., 2019; Moosavi et al., 2020; Selby et al., 2021; Glaspy et al., 2024).

Jinyouli^®^ was the first approved PEG-rhG-CSF for prophylaxis against FN in Chinese adult patients. Unlike adults, pediatric patients often undergo more intensive chemotherapy to achieve the desired antitumor effects, owing to fewer comorbidities. This may lead to severe myelosuppression (Wittman et al., 2006). A randomized clinical trial showed that pegfilgrastim was well-tolerated by young adults and children 1with sarcomas (Fox et al., 2009). However, no study focusing on PEG-rhG-CSF has been specifically designed for Chinese children. Therefore, a phase II clinical trial was conducted to assess the efficacy of PEG-rhG-CSF (Jinyouli^®^) in pediatric patients undergoing high-intensity chemotherapy. In addition to the previously reported interim results (Huang et al., 2023) this study reports the final results of this phase II trial.

## 2 Materials and methods

### 2.1 Study design

This open-label, multicenter, phase II study was conducted in 10 centers across China to evaluate the efficacy and safety of PEG-rhG-CSF in pediatric patients undergoing high-intensity chemotherapy. Data on children treated with rhG-CSF (Ruibai^®^) were retrospectively collected to establish an external control group for comparative reference in the pharmacoeconomic analysis, which was not pre-specified in the protocol.

This study was approved by the Ethics Committee of the Cancer Center of Sun Yat-sen University (approval number: B2020-202-01) and conducted according to the principles of the Declaration of Helsinki and Good Clinical Practices. Moreover, informed consent was obtained from each participant or their legal guardian. This study was registered with the Clinical Trial Registry (Trial Registration ID: NCT04547829).

### 2.2 Patient eligibility

Treatment-naïve pediatric patients (aged ≤18 years) with cytologically or histologically confirmed cancer were enrolled in the study. Patients scheduled to undergo high-intensity chemotherapy were eligible for inclusion. Additional inclusion criteria were as follows: a predicted survival of ≥8 months; an Eastern Cooperative Oncology Group performance status (ECOG PS) of 0–1; and normal bone marrow hematopoietic function (absolute neutrophil [ANC] ≥1.5 × 10^9^/L, platelet [PLT] ≥80 × 10^9^/L, hemoglobin [Hb] ≥75 g/L, and white blood cell [WBC] ≥3.0 × 10^9^/L counts).

The exclusion criteria included: uncontrolled local/systemic infection; severe visceral organ dysfunction (total bilirubin, alanine aminotransferase, and aspartate aminotransferase >2.5 × upper limit of normal [ULN] [>5 × ULN in patients with liver metastasis] and serum creatinine >2 × ULN); use of similar drugs or participation in other studies within 4 weeks before enrollment; allergy to PEG-rhG-CSF, rhG-CSF, or other preparations or proteins expressed by *Escherichia coli*; altered hematopoietic function after treatment; severe mental illness that might affect informed consent provision and/or adverse event (AE) observation; or unsuitability to participate in this study as judged by the investigators.

The external control cohort consisted of patients from real-world data sources who met the eligibility criteria for the study cohort.

### 2.3 Procedures

The study cohort underwent two cycles of high-intensity chemotherapy (each ≥14 days) as the primary cancer treatment. Patients subsequently received subcutaneous injections of 100 μg/kg PEG-rhG-CSF (Jinyouli^®^, CSPC Baike [Shandong] Biopharmaceutical Co., Ltd., Shandong, China) (total dose maximum 6 mg) as a primary prophylaxis within 24–48 h after each dose of chemotherapy. Subsequent chemotherapy was resumed at WBC > 2 × 10^9^/L, ANC > 0.8 × 10^9^/L, and PLT > 80 × 10^9^/L, allowing a 7-day recovery period for normalization where necessary. Patients who failed to meet these criteria during the recovery period were excluded from the study.

### 2.4 Assessments

Routine blood and biochemical analyses, routine urinalysis, 12-lead electrocardiography, and ECOG PS assessments were conducted 7 days before enrollment (baseline) and 1 day before chemotherapy initiation. Additionally, a routine blood examination was performed every other day following PEG-rhG-CSF treatment in each cycle until ANC was >0.5 × 10^9^/L. AEs were graded according to the National Cancer Institute Common Terminology Criteria for Adverse Events (NCI CTCAE) version 5.0 throughout the study. Additionally, safety assessments were performed in advance if the patients withdrew from the study because of toxic symptoms, withdrawal of consent, disease complications, or disease progression. Dose reductions and delays in chemotherapy were also recorded. We retrospectively collected total direct medical cost data from both the study group (PEG-rhG-CSF) and the external control group (rhG-CSF) for pharmacoeconomic analysis. The total direct medical cost data included drug, imaging, laboratory testing, bed, and other hospitalization expenses during the two inpatient chemotherapy cycles. All costs are presented in Chinese Yuan (RMB, ¥).

### 2.5 Endpoints

The primary endpoints were the incidence and severity of drug-related AEs and bone pain scores assessed using the Face, Legs, Activity, Cry, and Consolability (FLACC) scale or Wong–Baker facial pain rating scale (WBFPRS). Secondary endpoints included: incidence of grade 3–4 neutropenia (defined as ANC < 0.5 × 10^9^/L or 0.5–0.9 × 10^9^/L), duration of neutropenia recovery (ANC ≥ 1.8 × 10^9^/L), ANC changes, occurrence and duration of FN (defined as ANC < 0.5 × 10^9^/L and axillary temperature >38°C), nadir values of ANC, chemotherapy delays or dose reductions, proportion of patients administered antibiotics, and incidence and severity of AEs. The bone pain scores and AEs were assessed in all patients who received the study drug. The exploratory endpoint was the cost-effectiveness of PEG-rhG-CSF in Chinese children undergoing chemotherapy.

### 2.6 Statistical analyses

The sample size was calculated with 80% power to test the hypothesis that the incidence of bone pain would be 11% against the null hypothesis of 17% at a two-sided significance level (α) of 0.05. In total, 309 patients were included based on a 10% dropout rate.

Demographic characteristics were analyzed in the full analysis set (FAS) comprising patients who received at least one dose of the study drug. The safety analysis set (SS) included patients who received the study drug at least once and underwent at least one safety assessment.

The durations of neutropenia recovery were estimated using the Kaplan-Meier method to calculate median durations. ANC count for each cycle was descriptively summarized at different times and the nadir of neutropenia was calculated within each cycle. For chemotherapy delays or dose reductions, the proportion of patients administered antibiotics, and incidence and severity of AEs, data were descriptively summarized and presented as n (%) or median (range). For the pharmacoeconomic analysis, propensity-matching (PSM) ensured baseline data balance with a 3:1 nearest-neighbor ratio and a caliper width of 0.005, with age, sex, and weight as covariate factors.

Group comparisons were performed using the t-test, Wilcoxon rank-sum test, chi-square test, or Fisher’s exact test, as appropriate. The incremental cost-effectiveness ratio (ICER) was calculated, and the decision tree was modelled. Detailed statistical methods of the ICER are provided in Supplementary Methods S1, S2. One-way sensitivity analysis and the 1,000 Monte Carlo simulation probabilistic sensitivity analyses were performed to investigate the impact of varying alternative parametric assumptions on the ICER of the PEG-rhG-CSF and rhG-CSF groups.

All statistical analyses were performed using SPSS software (version 19.0; IBM Corp., Armonk, NY, United States) and STATA (version 14.0; Stata Corp., College Station, TX, United States). An α level of 0.05 was used for all statistical tests.

## 3 Results

### 3.1 Patient characteristics

**FIGURE 1:**
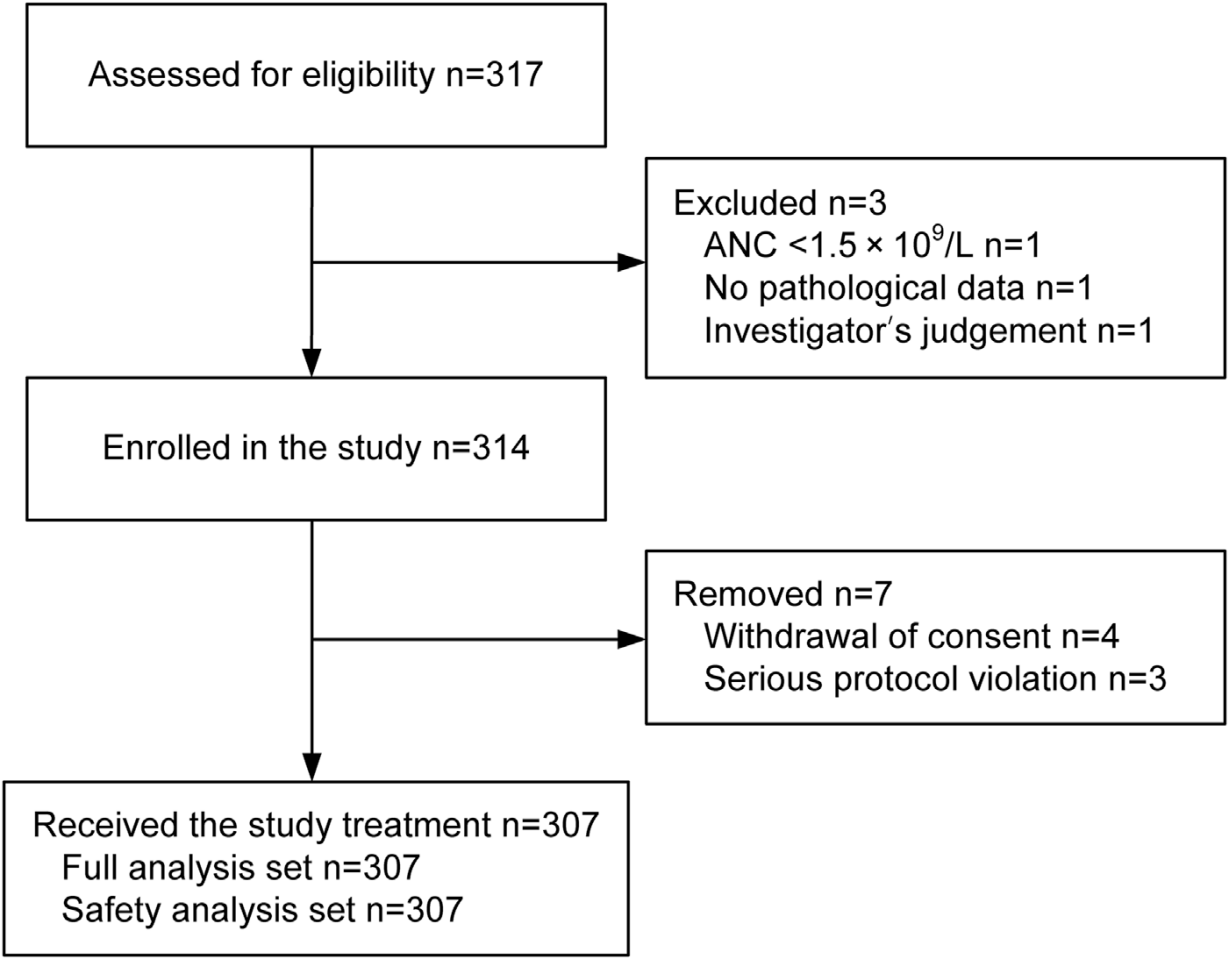
Patient enrollment and disposition.

Between October 2020 and October 2022, 317 patients were screened. Of these patients, three failed to meet the inclusion criteria for the following reasons: ANC < 1.5 × 10^9^/L (*n* = 1), absence of pathological data (*n* = 1), and investigator’s judgment (*n* = 1). Another seven patients were excluded from the study due to withdrawal of consent (*n* = 4) or serious protocol violation (*n* = 3). Thus, 307 patients received prophylactic PEG-rhG-CSF and were included in the FAS and SS (Figure 1). All included patients underwent one cycle and 288 (93.8%) underwent an additional cycle of PEG-rhG-CSF.

Patient demographics are shown in Table 1. The median patient age was 7.4 (range 0.3–18.1) years. Most patients had an ECOG PS of 1 (247/307, 80.5%) at baseline. In total, 134 patients (43.6%) had metastatic diseases. Of these, 64 (20.8%) had more than two metastatic sites.

**TABLE 1:** Baseline characteristics.

	Total (*n* = 307)
Age (years), median (range)	7.4 (0.3, 18.1)
Weight (kg), median (range)	21.4 (5.0, 96.5)
Sex, n (%)	
Male	171 (55.7)
Female	136 (44.3)
Baseline ANC (× 10 (Hu et al., 2021)/L), median (range)	3.8 (1.5, 18.2)
Baseline Hb (g/L), median (range)	118.0 (64.0, 175.0)
Baseline PLT (× 10 (Hu et al., 2021)/L), median (range)	354.0 (84.0, 768.0)
ECOG PS, n (%)	
0	36 (11.7)
1	247 (80.5)
2	18 (5.9)
Unknown	6 (2.0)
Disease, n (%)	
Sarcoma	114 (37.1)
Neuroblastoma	64 (20.8)
Lymphoma	41 (13.4)
Germ cell tumors	26 (8.5)
Brain tumors	32 (10.4)
Hepatoblastoma and others^a^	30 (9.8)
Number of organs involved in metastases, n (%)	
≤2	70 (22.8)
>2	64 (20.8)
Underlying diseases and associated comorbidities^b^, n (%)	34 (11.1)
Tumor-related medical history^c^, n (%)	13 (4.2)

ECOG PS, eastern cooperative oncology group performance status; ANC, absolute neutrophil count; PLT, platelet count; Hb, hemoglobin.

^a^ Other diseases included adrenocortical carcinoma and retinoblastoma.

^b^ Underlying diseases and associated comorbidities included thalassemia, glucose-6-phosphate dehydrogenase deficiency, epilepsy, nephrotic syndrome, *etc.*

^c^ Tumor-related medical history included extrarenal rhabdoid tumor and bone fibroma, *etc.*

### 3.2 Treatment

In total, 288 (288/307, 93.8%) patients completed two cycles of prophylactic PEG-rhG-CSF and chemotherapy. Supplementary Table S1 provides details of the chemotherapy regimens. Nineteen patients did not receive the second cycle of PEG-rhG-CSF because of voluntary withdrawal (*n* = 11), investigators’ decision (*n* = 4), AEs (*n* = 3), or protocol violations (*n* = 1).

### 3.3 Safety

**FIGURE 2:**
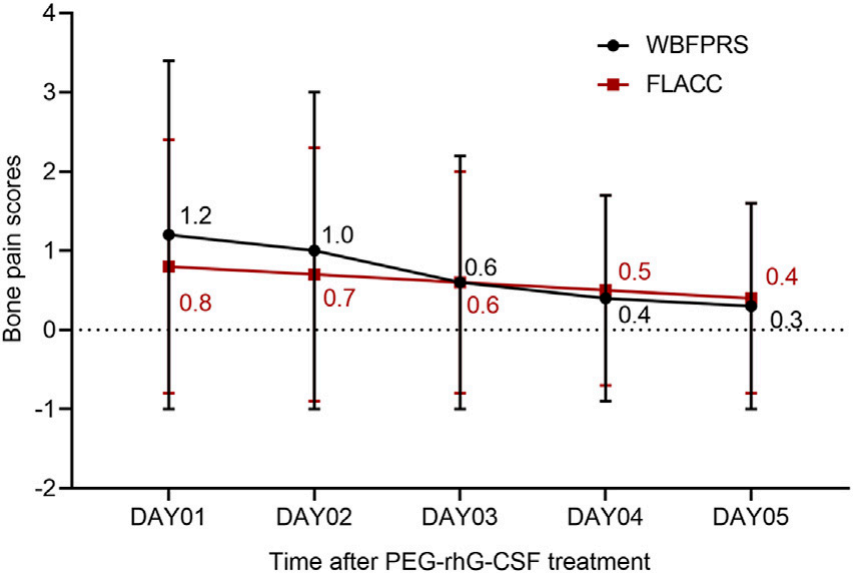
Bone pain scores FLACC, face, legs, activity, cry, and consolability; WBFPRS, Wong-Baker’s facial pain rating.

AEs of all grades occurred in all patients (Supplementary Table S2). Any-grade drug-related AEs were observed in 91 patients (29.6%), primarily including bone pain (39/307, 12.7%), myalgia (27/307, 8.8%), injection site reaction (27/307, 8.8%), malaise (22/307, 7.2%), fever (14/307, 4.6%), arthralgia (14/307, 4.6%), and dizziness (13/307, 4.2%). Most events (88/307, 28.7%), including bone pain (39/307, 12.7%), were grades 1–2. As shown in Figure 2, bone pain intensity peaked on Day 1 after treatment and subsequently decreased by 2-fold (FLACC score, 0.8 vs. 0.4; *n* = 322) or 4-fold (WBFPRS score, 1.2 vs. 0.3; *n* = 271) on Day 5. The bone pain scores of each patient at each cycle were rated independently. Grade 3 drug-related AEs included fever (1/307, 0.3), arthralgia (1/307, 0.3), and anaphylaxis (1/307, 0.3). No drug-related grade 4 AEs or deaths occurred. Moreover, drug-related AEs were more frequent in patients with sarcomas, lymphomas, and brain tumors, with bone pain predominantly observed in patients with sarcomas and lymphomas (Supplementary Table S3).

### 3.4 Efficacy

**FIGURE 3:**
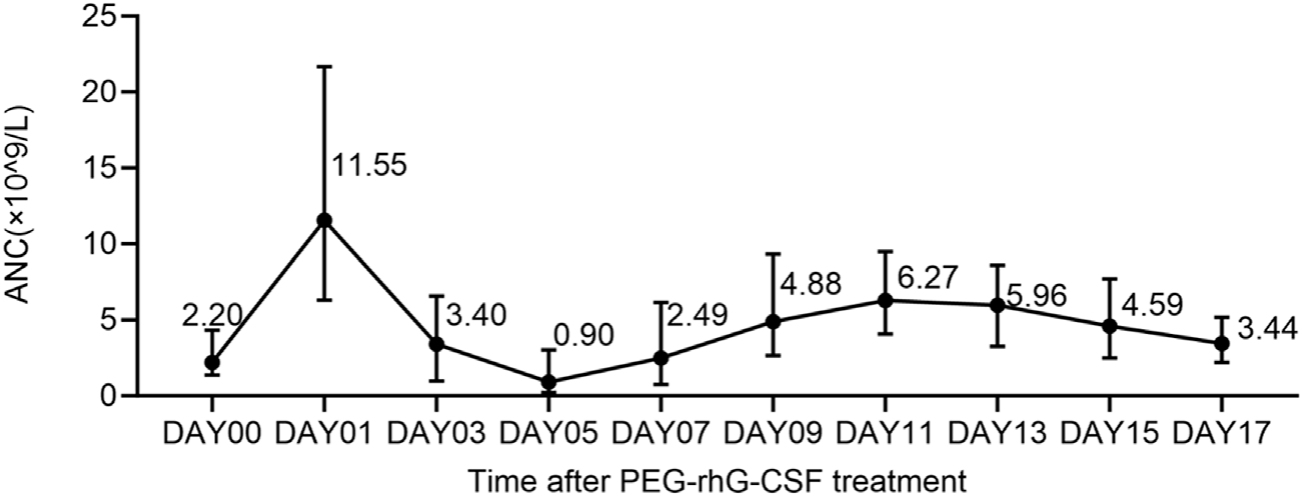
Median absolute neutrophil count after PEG-rhG-CSF ANC, absolute neutrophil count.

Grade 3–4 neutropenia was observed in Cycles 1 (70.7%, 217/307) and 2 (38.2%, 110/288). As shown in Figure 3, the median ANC peaked on Day 1, and the ANC nadir occurred on Day 5 after PEG-rhG-CSF treatment. The median duration of neutropenia recovery was 5.0 (range 1.0–29.0) and 4.0 (range 1.0–26.0) days in Cycles 1 and 2, respectively (Table 2). The median nadir value of ANC was higher in the second cycle (1.4 × 10^9^/L vs. 0.2 × 10^9^/L) than in the first cycle. Moreover, 123 (40.1%) and 27 (9.4%) patients in Cycles 1 and 2 experienced FN, with median durations of 3.0 (range 1.0–8.0) and 3.0 (range 1.0–11.0) days, respectively. Grade 3–4 neutropenia and FN occurred more frequently in patients with sarcomas, neuroblastomas, and lymphomas.

**TABLE 2:** Efficacy endpoint and related variables.

	Cycle 1 (*n* = 307)	Cycle 2 (*n* = 288)	Total (*n* = 307)
Grade 3–4 neutropenia, n (%)	217 (70.7)	110 (38.2)	239 (77.9)
Duration of neutropenia recovery^a^ (days), median (range)	5.0 (1.0, 29.0)	4.0 (1.0, 26.0)	-
FN, n (%)	123 (40.1)	27 (9.4)	132 (43.0)
Duration of FN (days), median (range)	3.0 (1.0, 8.0)	3.0 (1.0, 11.0)	-
Nadir values of ANC (× 10^9^/L), median (range)	0.2 (0.0, 12.7)	1.4 (0.0, 32.0)	0.1 (0.0, 9.3)
Chemotherapy dose reduction, n (%)	0	1 (0.3)	1 (0.3)
Chemotherapy dose delay, n (%)	0	2 (0.7)	2 (0.7)
Antibiotic therapy, n (%)	133 (43.3)	37 (12.8)	138 (45.0)

^a^ Duration of neutropenia recovery data were not available for one patient in cycle 1 and for 32 patients in cycle 2.

ANC, absolute neutrophil count; FN, febrile neutropenia.

No chemotherapy delays or reductions were observed in cycle 1. In contrast, two (0.7%) chemotherapy delays (one bed shortage and one grade 4 hydrocephalus) and one (0.3%) reduction in chemotherapy caused by grade 4 neutropenia were observed in cycle 2. During chemotherapy, antibiotics were administered to 138 patients (138/307, 45.0%) across 170 cycles, constituting 28.6% (170/595) of all cycles and 17.1% (29/170) for therapeutic use. Antibiotic administration in each cycle was rated independently for each patient. Table 2 shows the proportion of patients who received antibiotics during each cycle.

### 3.5 Pharmacoeconomic analysis

The Chemotherapy regimens of all 77 patients from the external control group were detailed in Supplementary Table S4. After PSM, 110 patients in the PEG-rhG-CSF study group and 74 in the rhG-CSF external control group were included in the pharmacoeconomic analysis. The baseline variables between the two groups were balanced (Supplementary Table S5). The mean total costs (¥12,911.77 vs. ¥17,218.57) of the PEG-rhG-CSF group were lower than those of the rhG-CSF group, with an incremental cost of -¥4,306.8. The effectiveness was 7.27% and 5.41% in the PEG-rhG-CSF and rhG-CSF groups, respectively, with an incremental effectiveness of 1.86%. These results indicate that PEG-rhG-CSF was a cost-effective strategy. The total hospitalization costs for the patients are provided in Supplementary Table S6.

**FIGURE 4:**
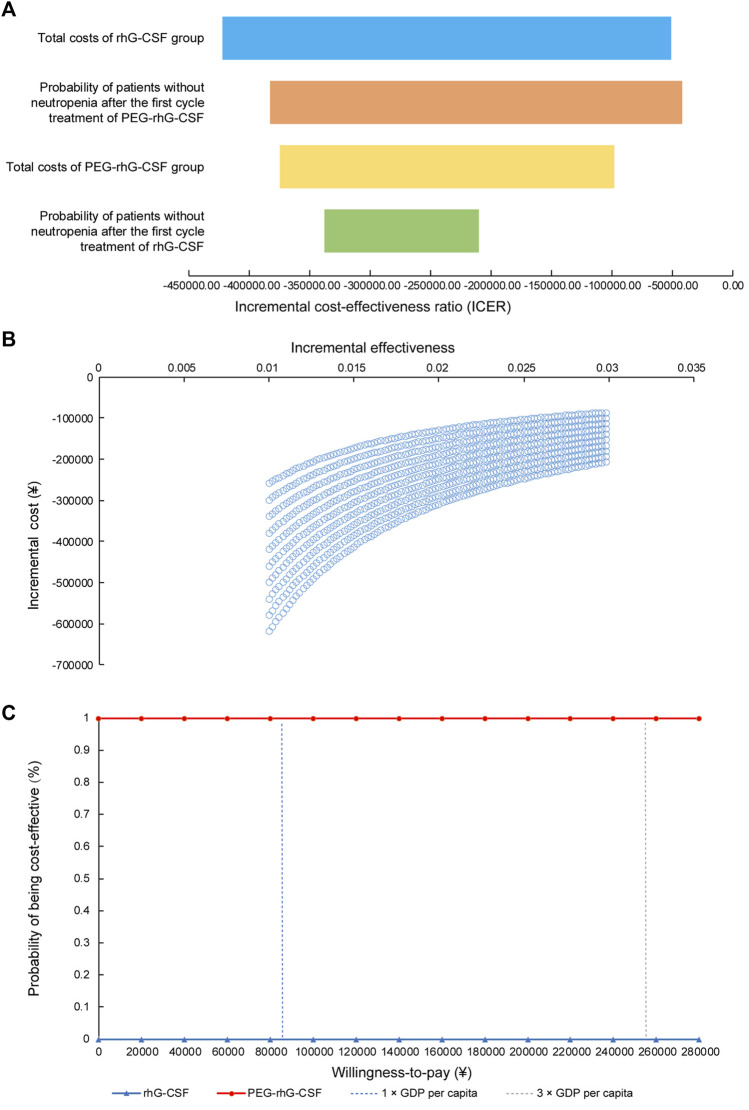
One-way sensitivity and probabilistic sensitivity analysis **(A)** Tornado diagram for one-way sensitivity analyses **(B)** Cost-effectiveness plane for PEG-rhG-CSF compared to rhG-CSF. The x-axis represented the difference in incremental cost-effectiveness and the Y-axis the difference in costs between PEG-rhG-CSF and rhG-CSF; **(C)** Cost-effectiveness acceptability curve.

Sensitivity analysis (Figure 4A) revealed that ICERs were most sensitive to total costs in the rhG-CSF group, followed by the probability of patients without neutropenia after the first cycle of PEG-rhG-CSF treatment. Furthermore, PEG-rhG-CSF was dominant in all simulations (Figure 4B, southeast quadrant). In the probabilistic sensitivity analysis (Figure 4C), the probability of PEG-rhG-CSF being cost-effective or dominant over the rhG-CSF prophylaxis strategy was 100% across a willingness-to-pay range of ¥0–¥260,000.

## 4 Discussion

To the best of our knowledge, this prospective, multicenter, phase II study is the first to investigate the efficacy of PEG-rhG-CSF (Jinyouli^®^) in Chinese pediatric patients treated with high-intensity chemotherapy. This drug exhibited low toxicity and promising efficacy, providing preliminary evidence to power a randomized controlled trial.

Both rhG-CSF and PEG-rhG-CSF induced the proliferation and maturation of neutrophils to reduce the incidence of FN, thereby presenting a therapeutic support strategy during intense-dose chemotherapy (Mei et al., 2022). PEG-rhG-CSF has a prolonged plasma half-life compared to unmodified rhG-CSF. Consistent with previous studies (Li et al., 2007; Spunt et al., 2010), this drug has shown efficacy and safety comparable to those of rhG-CSF in pediatric patients undergoing high-intensity chemotherapy. However, a single daily cycle of PEG-rhG-CSF provided considerable advantages over daily rhG-CSF administration for younger children, minimizing discomfort and distress associated with injections. Moreover, the administration of only one dose of PEG-rhG-CSF per chemotherapy cycle may enhance the quality of life of patients by reducing disruption to both patients and caregivers, potentially improving patient compliance. Considering the high risk of myelosuppression in children undergoing high-intensity chemotherapy, prophylactic therapy is preferred over salvage therapy, underscoring the benefits of long-acting PEG-rhG-CSF (Wittman et al., 2006). Unlike rhG-CSF, PEG-rhG-CSF does not require serial blood counts to determine the time to discontinue the procedure (Borinstein et al., 2009). Thus, daily PEG-rhG-CSF administration not only improved compliance but also mitigated the treatment burden on children and their families, potentially contributing to the accumulation of chemotherapeutic effects.

In the present study, PEG-rhG-CSF was well-tolerated by pediatric patients treated with high-intensity chemotherapy. Compared to prior studies on pediatric patients treated with pegfilgrastim, comparable incidences of grade 3 or higher AEs (82.7% vs. 84%) and drug-related AEs (29.6% vs. 22%) were observed in our study (Spunt et al., 2010). However, caution should be exercised regarding these comparisons because of the heterogeneity of tumors and chemotherapy regimens. The overall AEs observed were consistent with the known pharmacological effects of pegfilgrastim, with mild to moderate (grade 1–2) bone pain being the most common drug-related AE (André et al., 2007; Qin et al., 2017). Contrary to previous similar studies primarily focusing on the Caucasian population (0.67–21.0 years), the results from this study may offer valuable insights into the safety profile of PEG-rhG-CSF in younger (0.3–18.1 years) Asian pediatric patients (Spunt et al., 2010).

Pediatric patients administered with PEG-rhG-CSF exhibited a lower occurrence of FN (43.0% vs. 68%) and a shorter duration of neutropenia recovery during cycle 1 (5.0 vs. 14 days) than those who received pegfilgrastim (Spunt et al., 2010). This was potentially attributable to differences in tumor types and chemotherapy regimens between the two studies. Importantly, a substantial reduction in FN (9.4% vs. 40.1%) and antibiotic usage (12.8% vs. 43.3%) was observed during the second chemotherapy cycle compared with the first cycle, indicating the potential benefits of using PEG-rhG-CSF as a primary prophylaxis for reducing FN in pediatric patients undergoing chemotherapy. Additionally, other second-generation G-CSFs (such as mecapegfilgrastim, lipegfilgrastim, and empegfilgrastim) could consistently decrease the incidence of FN (Bondarenko et al., 2013; Bond et al., 2018; Xu et al., 2019). The probable explanation could be that second-generation G-CSFs can use their PEG moiety to stimulate granulocyte production and storage within the bone marrow while also facilitating the release of mature granulocytes, inducing both early and late peak release (You et al., 2023). No patient required a dose delay due to delayed recovery of blood counts in the present study. In addition, only one patient experienced a chemotherapy dose reduction due to neutropenia. Moreover, the absence of a high peak in the post-ANC nadir with PEG-rhG-CSF reflected self-regulatory receptor-mediated clearance (Fox et al., 2009). Overall, these findings demonstrate the efficacy of PEG-rhG-CSF (Jinyouli^®^) as a feasible prophylactic strategy for pediatric patients.

Consistent with previous studies, PEG-rhG-CSF was a cost-effective strategy in the present study (Fox et al., 2009). The reduced total costs (¥12,911.77 vs. ¥17,218.57) of PEG-rhG-CSF compared to rhG-CSF may be attributed to lower FN occurrence, shorter duration of ANC recovery, and lower antibiotic usage. Our analysis focused on the viewpoint of the payer. However, from a societal perspective, the cost-effectiveness of PEG-rhG-CSF per chemotherapy cycle may also be improved over daily rhG-CSF, given the reduced patient consultation time, caregiver expenses, and lost productivity (Weinstein et al., 1996; Akpo et al., 2017). Despite being an essential decision-making tool, few pharmacoeconomic studies are available for pediatric patients, making this study valuable for clinical reference in this regard.

This study had some limitations. The small sample size and single-arm design without a control group for comparison posed a challenge to decipher the benefits of PEG-rhG-CSF treatment. The retrospective data for pharmacoeconomic analysis may introduce bias. Therefore, further randomized controlled trials are required to confirm our findings. Second, the heterogeneous population of this study potentially introduced unmeasured confounding variables, highlighting the need for larger studies to further elucidate clinical decisions in heterogeneous cancer types. Third, the relatively long observation period and its retrospective nature may have biased the results of the pharmacoeconomic analysis.

This study demonstrated the efficacy, safety, and cost-effectiveness of prophylactic PEG-rhg-CSF in 307 pediatric Chinese patients undergoing high-intensity chemotherapy. Here, PEG-rhg-CSF exhibited low toxicity and promising efficacy. Furthermore, it effectively reduced the incidence of FN. Thus, PEG-rhG-CSF (Jinyouli^®^) may provide a convenient, safe, efficient, and cost-effective option for primary prophylaxis of myelosuppression in pediatric patients undergoing high-intensity chemotherapy.

## Data Availability

The datasets presented in this study can be found in online repositories. The names of the repository/repositories and accession number(s) can be found below: https://www.researchdata.org.cn/.
