# Supplementary material for: Efficacy, safety, and cost-effectiveness of pegylated PEG-rhg-CSF in pediatric patients receiving high-intensity chemotherapy: results from a phase II study
Source: Front Pharmacol. 2024 Jul 17;15:1419369. doi: 10.3389/fphar.2024.1419369 (PMC11288831; doi:10.3389/fphar.2024.1419369)
Supplement: Supplementary file 1 [file DataSheet1.docx]

Supplementary Material

# Supplementary Methods

## Method S1. Decision tree model

**

**

## Method S2. Cost-effectiveness analysis

Cost data were converted to the total inpatient cost for the year 2022 using the Healthcare & Medical Service of China Consumer Price Index (CPI). The Healthcare & Medical Service of China CPIs from 2010 to 2022 were sourced from the National Bureau of Statistics of China (https://data.stats.gov.cn/). Cost data for inpatients in 2023 were discounted at a rate of 5% and converted to 2022 data. Cost data were adjusted for inflation to 2022 values using the CPI for all consumers, using 2009 as a baseline of 100. As an example, inpatient costs in 2009 were converted to CPI-adjusted 2022 dollars by first multiplying costs by the 2022 CPI of 100.6 and then dividing by the 2009 CPI of 100.

Incremental cost-effectiveness ratio (ICER) was calculated using the following formula: ICER= (C_J_ - C_R_)/ [(100% - I_J_) -(100% - I_R_)], C_J_ represented the average total inpatient cost per patient in PEG-rhG-CSF group, C_R_ represented the average total inpatient cost per patient in rhG-CSF group, I_J_ represented the incidence of neutropenia in PEG-rhG-CSF group, I_R_ represented the incidence of neutropenia in rhG-CSF group. According to China Guidelines for Pharmacoeconomic Evaluations (2020), if the ICER is equal to or less than 1 × Gross Domestic Product (GDP) per capita then the intervention is deemed highly cost effective and if the ICER is between 1 and 3 × GDP per capita then the intervention is considered cost effective. Above 3 × GDP per capita, the intervention is considered not cost effective.

| **Years** | **China Consumer Price Index (CPI): Healthcare & Medical Service**  **(last year=100)** |
| --- | --- |
| **2010** | 103.3 |
| **2011** | 102.9 |
| **2012** | 101.7 |
| **2013** | 101.5 |
| **2014** | 101.7 |
| **2015** | 102.7 |
| **2016** | 103.8 |
| **2017** | 106.0 |
| **2018** | 104.3 |
| **2019** | 102.4 |
| **2020** | 101.8 |
| **2021** | 100.4 |
| **2022** | 100.6 |

# Supplementary Tables

## Table S1. Treatment exposure

|  | **All patients (n=307)** |
| --- | --- |
| **Chemotherapy regimens** |  |
| CAV/IE | 65 (21.2) |
| CAV/VIP | 37 (12.1) |
| SCCCG-BL | 24 (7.8) |
| CAV/CE | 22 (7.2) |
| EP/IE | 14 (4.6) |
| JEB | 14 (4.6) |
| VAC/VII | 13 (4.2) |
| VAC | 12 (3.9) |
| Others | 106 (34.5) |
| **PEG-rhG-CSF** **administration** |  |
| Cycle 1 | 307 (100.0) |
| Cycle 2 | 288 (93.8) |
| **Concomitant rhG-CSF** | 110 (35.8) |
| Cycle 1 | 106 (34.5) |
| Cycle 2 | 28 (9.7) |

CAV/IE, cyclophosphamide + pirarubicin + vincristine/ifosfamide + etoposide; CAV/VIP, cyclophosphamide + pirarubicin + vincristine/ifosfamide + etoposide + cisplatin; SCCCG-BL, a modified BFM protocol for non-Hodgkin lymphoma; CAV/CE, cyclophosphamide + pirarubicin + vincristine/etoposide + carboplatin; JEB, etoposide + carboplatin + bleomycin; VAC/VII, cyclophosphamide + actinomycin-D + vincristine/ifosfamide + irinotecan; VAC, vincristine + actinomycin-D + cyclophosphamide.

## Table S2. Drug-related adverse events by tumor types

|  | **Sarcoma**  **(n=114)** | **Neuroblastoma**  **(n=64)** | **Lymphoma**  **(n=41)** | **Germ-cell tumor**  **(n=26)** | **Brain tumor**  **(n=32)** | **Others**  **(n=30)** |
| --- | --- | --- | --- | --- | --- | --- |
| **All drug-related AEs** | 108 (94.7) | 31 (48.4) | 38 (92.7) | 18 (69.2) | 25 (78.1) | 16 (53.3) |
| Bone pain | 25 (21.9) | 9 (14.1) | 11 (26.8) | 2 (7.7) | 2 (6.3) | 3 (10.0) |
| Injection site reaction | 13 (11.4) | 1 (1.6) | 4 (9.8) | 1 (3.8) | 8 (25.0) | 5 (16.7) |
| Myalgia | 15 (13.2) | 2 (3.1) | 9 (22.0) | 2 (7.7) | 1 (3.1) | 2 (6.7) |
| Malaise | 18 (15.8) | 2 (3.1) | 4 (9.8) | 1 (3.8) | 2 (6.3) | 0 |
| Fever | 3 (2.6) | 7 (10.9) | 2 (4.9) | 2 (7.7) | 2 (6.3) | 0 |
| Arthralgia | 8 (7.0) | 0 | 4 (9.8) | 2 (7.7) | 0 | 2 (6.7) |
| Dizziness | 10 (8.8) | 1 (1.6) | 1 (2.4) | 3 (11.5) | 1 (3.1) | 0 |
| Vomiting | 3 (2.6) | 3 (4.7) | 0 | 0 | 1 (3.1) | 3 (10.0) |
| Abdominal pain | 3 (2.6) | 1 (1.6) | 0 | 0 | 0 | 0 |
| Allergic rash | 1 (0.9) | 1 (1.6) | 1 (2.4) | 0 | 1 (3.1) | 0 |
| Nausea | 1 (0.9) | 0 | 0 | 1 (3.8) | 1 (3.1) | 0 |
| Toothache | 1 (0.9) | 1 (1.6) | 0 | 0 | 1 (3.1) | 0 |
| Injection site induration | 1 (0.9) | 0 | 0 | 0 | 2 (6.3) | 0 |
| Tachypnea | 0 | 0 | 0 | 2 (7.7) | 0 | 0 |
| Muscle cramp | 0 | 0 | 0 | 0 | 1 (3.1) | 1 (3.3) |
| Lymphocyte count decreased | 1 (0.9) | 1 (1.6) | 0 | 0 | 0 | 0 |
| Facial pain | 1 (0.9) | 1 (1.6) | 0 | 0 | 0 | 0 |
| Insomnia | 1 (0.9) | 0 | 0 | 1 (3.8) | 0 | 0 |
| Acrodynia | 2 (1.8) | 0 | 0 | 0 | 0 | 0 |
| Neutrophil count decreased | 0 | 0 | 0 | 1 (3.8) | 1 (3.1) | 0 |
| Leukocytosis | 0 | 0 | 1 (2.4) | 0 | 0 | 0 |
| Diarrhea | 1 (0.9) | 0 | 0 | 0 | 0 | 0 |
| Cough | 0 | 1 (1.6) | 0 | 0 | 0 | 0 |
| Anaphylactic shock | 0 | 0 | 0 | 0 | 1 (3.1) | 0 |
| Headache | 0 | 0 | 1 (2.4) | 0 | 0 | 0 |

## Table S3. Incidence of grade 3-4 neutropenia and febrile neutropenia by tumor type

|  | **Sarcoma**  **(n=114)** | **Neuroblastoma**  **(n=64)** | **Lymphoma**  **(n=41)** | **Germ-cell tumor**  **(n=26)** | **Brain tumor**  **(n=32)** | **Others**  **(n=30)** |
| --- | --- | --- | --- | --- | --- | --- |
| **Grade 3-4 neutropenia, n (%)** | | | | | | |
| Cycle 1 | 83 (72.8) | 53 (82.8) | 35 (85.4) | 13 (50.0) | 14 (43.8) | 19 (63.3) |
| Cycle 2 | 33 (30.3) | 31 (50.0) | 20 (48.8) | 3 (12.0) | 11 (44.0) | 12 (46.2) |
| Total | 91 (79.8) | 57 (89.1) | 35 (85.4) | 15 (57.7) | 20 (62.5) | 21 (70.0) |
| **Febrile neutropenia, n (%)** | | | | | | |
| Cycle 1 | 52 (45.6) | 32 (50.0) | 20 (48.8) | 5 (19.2) | 7 (21.9) | 7 (23.3) |
| Cycle 2 | 8 (7.3) | 9 (14.5) | 6 (14.6) | 0 | 3 (12.0) | 1 (3.8) |
| Total | 55 (48.2) | 34 (53.1) | 21 (51.2) | 5 (19.2) | 9 (28.1) | 8 (26.7) |

## Table S4. Treatment exposure in external control group

|  | **All patients (n=77)** |
| --- | --- |
| **Chemotherapy regimens** |  |
| CAV/EP | 18 (23.4) |
| BFM95 | 12 (15.6) |
| CAV/IE | 12 (15.6) |
| PEB | 12 (15.6) |
| VAC | 6 (7.8) |
| DDP+CCNU+VCR | 3 (3.9) |
| ABVD | 2 (2.6) |
| AP | 2 (2.6) |
| C5V | 2 (2.6) |
| CEV | 2 (2.6) |
| IE | 2 (2.6) |
| BEACOPP | 1 (1.3) |
| EP | 1 (1.3) |
| VIDE | 1 (1.3) |
| VIP | 1 (1.3) |

CAV/EP: Cyclophosphamide + Doxorubicin/Pirarubicin + Vincristine followed by Etoposide + Platinum (cis- or carboplatin); CAV/IE, Cyclophosphamide + Pirarubicin + Vincristine/Ifosfamide + Etoposide; BFM95, protocol for lymphoma inclusive of Prednisone, Vincristine, Anthracycline, Methotrexate, Cyclophosphamide, and others; PEB, Prednisone + Etoposide + Bleomycin; VAC, Vincristine + Actinomycin D + Cyclophosphamide; DDP+CCNU+VCR, Cisplatin + Carmustine + Vincristine; ABVD, Doxorubicin + Bleomycin + Vinblastine + Dacarbazine; AP, Doxorubicin + Prednisone; C5V, Cyclophosphamide + Adriamycin + Vincristine + Vinblastine; CEV, Cyclophosphamide + Etoposide + Vincristine; IE, Ifosfamide + Etoposide; BEACOPP, Bleomycin + Etoposide + Doxorubicin + Cyclophosphamide + Vincristine + Procarbazine + Prednisone; VIDE, Vincristine + Ifosfamide + Doxorubicin + Etoposide; VIP, Vincristine + Ifosfamide + Cisplatin.

## Table S5. Baseline characteristics after propensity-matching

|  | **PEG-rhG-CSF group (n=110)** | **rhG-CSF group (n=74)** | **t/χ^2^** | **P** |
| --- | --- | --- | --- | --- |
| **Age (years), median (IQR)** | 4 (2, 7) | 3.0 (2, 5.0) | 1.828 | 0.176 |
| **Sex (n, %)** |  |  | 1.008 | 0.315 |
| Male | 71 (64.55) | 53 (71.62) |  |  |
| Female | 39 (35.45) | 21 (28.38) |  |  |
| **Weight (kg), median (IQR)** | 15.2 (12.0, 22.3) | 13.0 (12.0,18.0) | 2.74 | 0.098 |
| **Diseases, n (%)** |  |  |  |  |
| Neuroblastoma | 31 (28.18) | 19 (25.68) | 0.14 | 0.708 |
| Burkitt lymphoma | 2 (1.82) | 9 (12.16) | 8.42 | 0.004 |
| Rhabdomyosarcoma | 27 (24.55) | 12 (16.22) | 1.84 | 0.175 |
| Yolk sac tumor | 4 (3.64) | 4 (5.41) | 0.33 | 0.564 |
| Hepatoblastoma | 5 (4.55) | 5 (6.76) | 0.42 | 0.516 |
| Hodgkin lymphoma | 1 (0.91) | 5 (6.76) | 4.80 | 0.029 |
| Ewing's sarcoma | 4 (3.64) | 0 | 2.69 | 0.101 |
| Diffuse large B-cell lymphoma | 0 | 2 (2.70) | 3.01 | 0.083 |
| Others | 33 (30.00) | 15 (20.27) | 2.17 | 0.141 |
| **Treated with high-intensity chemotherapy regimen, n (%)** | 110 (100.00) | 74 (100.00) | NA | NA |
| **Normal Bone marrow hematopoietic function at initial diagnosis, n (%)** | 110 (100.00) | 74 (100.00) | NA | NA |

IQR, interquartile range; SD, standard deviation; NA, not applicable.

## Table S6. Costs of treatments after propensity-matching

|  | **PEG-rhG-CSF group (n=110)** | **rhG-CSF group (n=74)** | **P** |
| --- | --- | --- | --- |
| **Without neutropenia in cycle 1 (¥,** **mean ± SD)** |  |  |  |
| Drug withdrawal after cycle 1 | NA | 7433.67 ± 7513.62 | NA |
| Without neutropenia in cycle 2 | 12500.28 ± 8817.86 | 31437.67 ± 34883.65 | 0.145 |
| With neutropenia and no fever in cycle 2 | 6618.81 ± 3442.65 | 16603.38 ± 9104.08 | 0.007 |
| With neutropenia and fever in cycle 2 | NA | 24007.67 ± 18943.94 | NA |
| **With neutropenia and no fever in cycle 1 (¥,** **mean ± SD)** |  |  |  |
| Drug withdrawal after cycle 1 | 4904.40 ± 1562.55 | 8094.31 ± 7837.81 | 0.589 |
| Without neutropenia in cycle 2 | 23749.89 ± 43398.02 | 19805.18 ± 9659.82 | 0.845 |
| With neutropenia and no fever in cycle 2 | 11592.85 ± 13590.53 | 18158.83 ± 12808.55 | 0.176 |
| With neutropenia and fever in cycle 2 | 11455.17 ± 13233.55 | 13964.65 ± 9108.14 | 0.826 |
| **With neutropenia and fever in cycle 1 (¥,** **mean ± SD)** |  |  |  |
| Drug withdrawal after cycle 1 | 5611.55 ± 3682.59 | 12983.03 ± 10062.95 | 0.157 |
| Without neutropenia in cycle 2 | 12925.43 ± 11552.22 | 24449.37 ± 15087.49 | 0.223 |
| With neutropenia and no fever in cycle 2 | 11017.57 ± 5591.44 | 19307.06 ± 9724.83 | 0.018 |
| With neutropenia and fever in cycle 2 | 19053.22 ± 11274.93 | 25903.52 ± 22499.89 | 0.548 |

SD, standard deviation; NA, not applicable.
